# A case of anti- *pityriasis versicolor* therapy that preserves healthy mycobiome

**DOI:** 10.1186/s12895-020-00106-x

**Published:** 2020-09-29

**Authors:** Mariusz Dyląg, Ewa Leniak, Sebastian Gnat, Jacek C. Szepietowski, Lukasz Kozubowski

**Affiliations:** 1 Department of Mycology and Genetics, Institute of Genetics and Microbiology, University of Wroclaw, Wroclaw, Poland; 2 Max Planck Institute of Molecular Plant Physiology, 14476 Potsdam, Germany; 3 Department of Veterinary Microbiology, Institute of Biological Bases of Animal Diseases, Faculty of Veterinary Medicine, University of Life Sciences in Lublin, Lublin, Poland; 4 Department and Clinic of Dermatology, Venereology and Allergology Wroclaw Medical University, Wroclaw, Poland; 5 Department of Genetics and Biochemistry, Clemson University, Clemson, SC USA

**Keywords:** *Malassezia*, Hyperpigmentation, Hypopigmentation, Dysbiosis, Treatment

## Abstract

**Background:**

The impact of Malassezia yeasts on skin mycobiome and health has received considerable attention recently. *Pityriasis versicolor* (PV), a common dermatosis caused by Malassezia genus worldwide, is a manifestation of dysbiosis. PV can be associated with hyper- and/or hypopigmented skin lesions. This disease entity is characterized by high percentage of relapses, which demands a proper antifungal therapy that is based on unambiguous species identification and drug susceptibility testing.

**Case presentation:**

Comprehensive analysis of PV case in man presenting simultaneously hyper- and hypopigmented skin lesions was performed. Conventional and molecular diagnostic procedures revealed *Malassezia furfur* and *Malassezia sympodialis,* respectively as etiological agents of skin lesions observed. Susceptibility tests showed significantly lowered sensitivity of *M. furfur* cells to fluconazole. Based on susceptibility profiles local antifungal therapy with drugs characterized by entirely different mechanism of action was included.

**Conclusions:**

Our study indicates that cases of PV represented by two types of skin lesions in one patient may be associated with distinct Malassezia species. Moreover, as observed in this case, each of the isolated etiological agents of PV may differ significantly in susceptibility to antifungals. This can significantly complicate the treatment of dermatosis, which by definition is associated with a significant percentage of relapses. In the presented case localized topical treatment was sufficient and successful while allowing maintaining the physiological mycobiome.

## Background

*Pityriasis versicolor* (PV) also known as *tinea versicolor* is caused by basidiomycetous yeasts of the genus *Malassezia*, characterized by lipid-dependent growth [1–3] and narrow specialization in the context of occupied ecological niches [2]. With respect to human skin, species of the genus *Malassezia* may have positive as well as negative health impact [4–6]. These lipophilic fungi are the most important components of human skin mycobiome [6–8]. It is widely accepted that the composition of the skin microbiome is critical for its health and condition [6, 7]. *Malassezia restricta*, *Malassezia globosa* and *Malassezia sympodialis* are the most often isolated species from the skin of healthy individuals [8–11]. Under certain conditions, however these *Malassezia* species, similar to other opportunistic pathogens, break out of the internal control of the host and overgrow leading to skin infections [5]. While on healthy skin *Malassezia* proliferates as budding yeast, during an outbreak state cells often develop into pseudohyphae leading to the development of typical symptoms associated with PV [5, 12]. Places on the human body particularly predisposed to the development of the above-mentioned disease include skin areas rich in sebum (source of triglycerides) produced by sebaceous glands [13]. Such pathomechanism of infection is most typical for *M. globosa* and *M. furfur*, but can also involve *M. sympodialis* which are able to form pseudohyphae in human stratum corneum [14, 15] and are the most common etiological factors of PV worldwide [4, 16, 17]. PV is one of the most common superficial fungal infections, although does not pose a serious threat to the health or life of the patient [4, 12, 18]. PV is not accompanied by pain, and inflammation; itching, and exfoliation of the skin are rather mild [5, 18]. In most cases, PV poses a problem mainly of an aesthetic nature, due to the accompanying skin discoloration, taking the form of hyper- or hypopigmentation, the latter known as PV *alba* or PVa [19]. However, PV may be characterized by high percentage of relapses [19, 20], especially among residents of tropical regions [17, 20], or because of improper therapy [21]. PV also stands out from other fungal infections in the context of the spectrum of people predisposed to the development of this disease. Among the most important internal and external risk factors promoting development of PV are oily skin (rich in sebum), impaired hormonal balance, hyperhidrosis, administration of corticosteroids or broad-spectrum antibiotics, genetic predispositions, and high temperature and relative humidity that prevail in tropical and subtropical regions [5, 18, 22]. Unlike most of known fungal diseases, superficial infections caused by *Malassezia* yeasts most often affect adults in their prime of life, who are usually not inflicted by any serious underlying diseases [5, 23], what was also demonstrated in our previous study [24].

In this work, we describe PV case involving two various etiological factors of infection underlying two different clinical manifestations in one patient. In the case presented, *M. furfur* (strain Mf_MD2) and *M. sympodialis* (strain Msy_MD10) were isolated from hypopigmented and hyperpigmented skin lesions, respectively. The third strain, *M. restricta* (Mr_MD2) was isolated only from healthy skin around the neck and face of the patient, free of any visible skin lesions. Furthermore, in control examination of healthy skin of patient’s wife the same *M. sympodialis* as well *M. restricta* isolates, but not *M. furfur* was isolated. Based on clinical manifestations of the disease, examination in light of Wood lamp, and replicable results of conventional and molecular tests, we were able to deliver evidences on etiology of two clinical types of skin lesions. Importantly, this work demonstrates that use of topical antifungal drugs, applied only to affected skin, is sufficient to cure PV and simultaneously preserve skin mycobiome.

## Case presentation

**Fig. 1:**
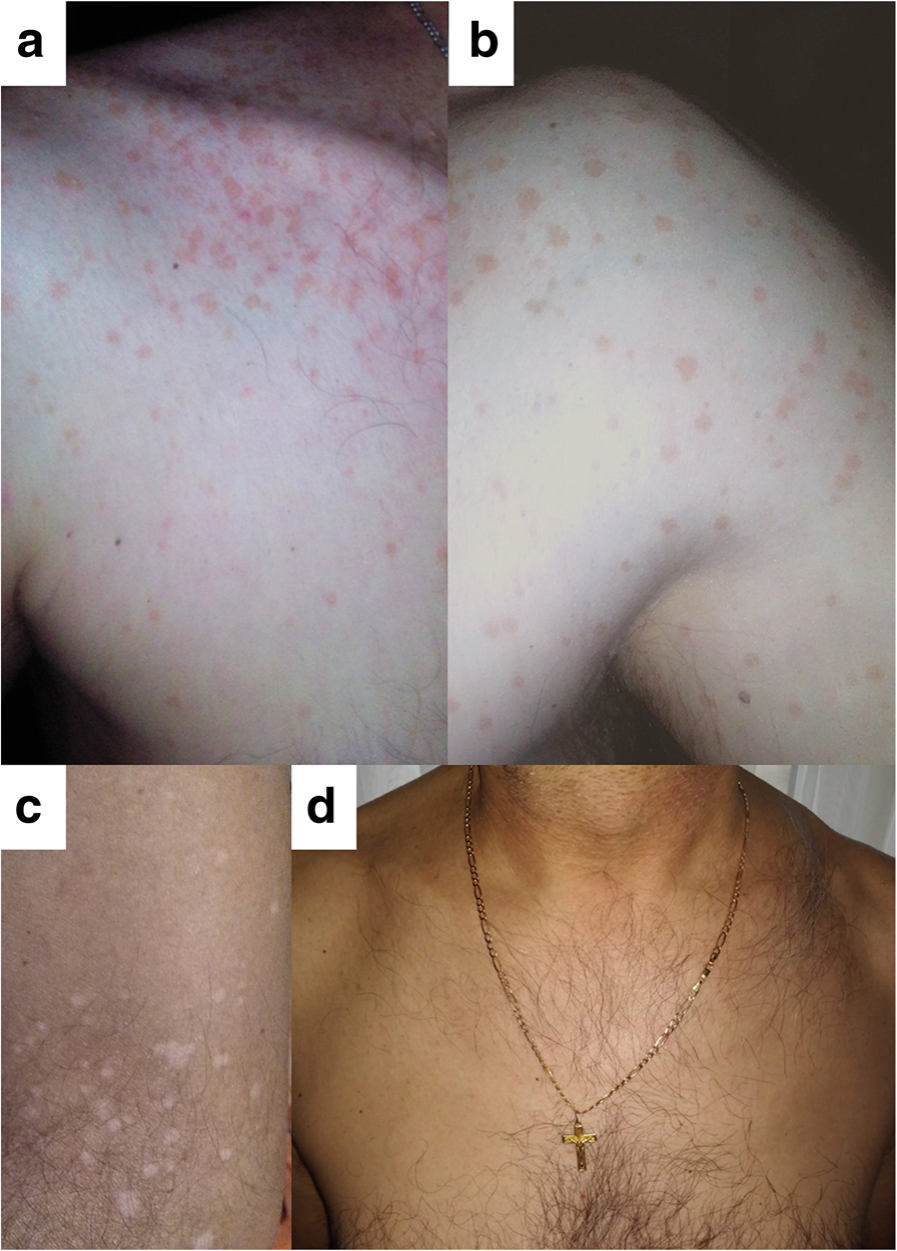
Clinical picture of affected (**a**-**c**) (*pityriasis versicolor*) and healthy (**d**) skin (after therapy) in man. Hyperpigmented (**a** and **b**) and hypopigmented (**c**) skin lesions of different etiology are indicated

In October 2019, a 50-year-old fair complexion man, resident of Lower Silesia (Poland), construction manager by profession, after dermatological consultation was directed for a mycological examination to the local laboratory. The first skin changes patient observed on chest in May 2019 and then disease manifestations developed systematically. In an interview, the patient revealed that in August 2019, with no prior medical consultation, he administered an antifungal cream containing 0.5% fluconazole. According to the patient the treatment was irregular (i.e. two times per week for 2 weeks) and for this reason with no visible improvement. During the visit to the dermatologist, basic physical examinations and those using the Wood lamp and dermatoscope were carried out. On the day of consultation, extensive hyper- and hypopigmented macules on patient’s skin were diagnosed that were characterized by and with an uneven edges, moderate skin scaling and the accompanying mild itching, clearly visible when using a dermatoscope. Skin lesions covered the chest, shoulders, back, arms and forearms (Fig. 1a-c). Patches were not spilled, but took the form of numerous small beige discolorations, with the exception of forearms where patches were white and rather resembled *vitiligo* (Fig. 1c). It should also be noted that forearms, due to sunlight exposure during many hours of construction work in the field, were clearly tanned, unlike other parts of the body where hyperpigmented macules were found. The patient was immunocompetent and in overall good health. Medical records and an interview indicated that neither the patient nor his family had any systemic, psychosomatic or chronic skin diseases. The patient had no prior history of immunosuppressive drugs treatments. Based on characteristic symptoms PV was diagnosed. The decision on the type of antifungal therapy was made based on the characteristic clinical symptoms of the disease, and the results of direct mycological examination, and susceptibility tests. Despite the fact that the skin lesions were quite widespread, oral treatment with systemic antifungal drugs was not included. This allowed to avoid interference with the composition of the gastrointestinal microbiome, and minimized disturbance of the mycobiome of healthy skin. After obtaining patient approval, at the beginning of November 2019, based on the results of the in vitro susceptibility tests, the therapy was set as follows: topical administration of ciclopirox 1% cream once daily (applied overnight), and terbinafine 1% emulsion gel applied once daily (in the morning) for 14 and 7 days, respectively. Direct microscopy examination of the skin scrapings, collected from hyper- and hypopigmented macules, performed 2 weeks after completed treatment, still revealed the presence of some fragments of pseudohyphae and single degenerated cells, while the results of cultures on modified Leeming-Notman agar (MLNA) were negative. In contrast to affected skin, biological material scraped from healthy skin around the neck and face contained *M. restricta* and *M. sympodialis*. The last mycological examination combined with consultation with a dermatologist, performed 2 months after completed therapy, has confirmed eradication of the infection evidenced both by microscopy and in cell culture. Some changes in skin pigmentation, what was anticipated, remained present mainly in the hypopigmented areas. However, 4 months after the end of the therapy, white discoloration was resolved, according to the patient’s feedback. Additionally, after this time, results of physical examination were normal and blood tests were within normal limits. Finally, the patient experienced no relapse on the day of publication, exactly during 8 months follow-up period (Fig. 1d). This led to the conclusion that treatment has been fully successful. On the other hand, direct examination and positive culture on MLNA, carried out for control purposes, revealed the presence of viable single budding cells of *M. restricta* and *M. sympodialis* in examined areas of patient’s healthy skin. For comparison (in accordance with the wishes of the patient and his wife), taking into account epidemiological dependencies and in the context of healthy skin mycobiome, diagnostic material was also collected from skin around the chest and neck of patient’s 49-year-old wife.

Methods and results of conventional and molecular mycological diagnostic procedures leading to unambiguous identification of both etiological factors of PV as lipid-dependent components of the skin mycobiome are included as an integral part of this study (see Additional file 1).

## Discussion and conclusions

**Fig. 2:**
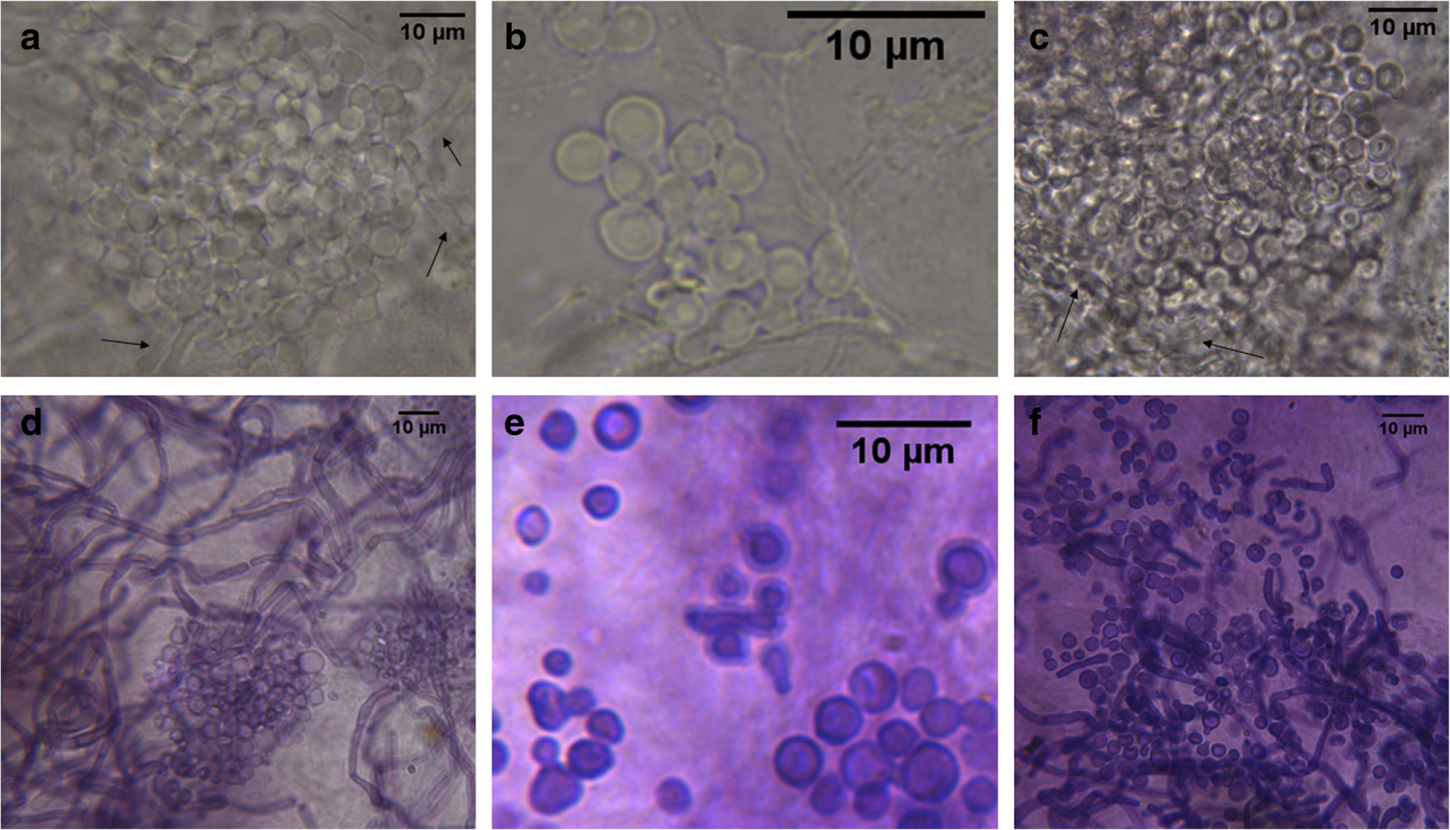
Histological patterns of fungal structures observed in skin scrapings. Skin scales taken from hypopigmented (**a, d**) and hyperpigmented (**c, f**) lesions, and for control from healthy skin around the neck and face of man (**b, e**). Microscopic preparations in DMSO with 10% KOH (**a**-**c**) or stained by PAS technique (**d**-**f**), magnification: **a** and **c** 1000x, **d** and **f** 400x, **e** 2000x, and **b** 3000x

PV is most commonly manifested as hyperpigmented and/or hypopigmented skin lesions [5, 25–27]. A direct examination of skin scales from these lesions typically reveals abundant pseudohyphae and clusters of cells in a characteristic,spaghetti with meatballs” pattern [18], also observed in this study (Fig. 2a, c, d, f). In contrast to affected skin, pseudohyphae are typically absent in the material collected from healthy skin [5, 28], consistent with the case described here (Fig. 2b, e).

The coexistence of hypo- and hyperpigmented skin lesions has been described elsewhere [29–31]. However, in many of such cases there is no information if one or two etiological agent(s) caused these distinct lesions [30, 31]. Until the study reported by Gaitanis et al. [26], only one etiological factor of *Malassezia* genus has been perceived as associated with PV, regardless of the types of symptoms accompanying the disease. The study by Gaitanis et al. has provided an explanation for the dual nature of the etiology of hypo- and hyperpigmented skin lesions examined in six female patients [26]. However, the exact mechanisms leading to development of hyper- and hypopigmented lesions has not yet been fully elucidated, and several plausible explanations have been proposed [32–34]. The most probable seems the hypothesis pointing to dissimilarities in the ability to synthesize melanin by *M. furfur* and *M. sympodialis*, first time formulated by Gaitanis et al. [26] and later explained by Ianirii et al. based on molecular tools [35]. One of the causes of depigmenting effect, observed also in case of our patient on tanned skin of forearms and lower parts of arms (Fig. 1c), could be azelaic acid, which inhibits the melanogenesis process [32].

In the case study presented here, skin scrapings from two types of skin lesions in a single patient were collected separately and treated individually, as if the biological material originated from two separate disease entities. Such a diagnostic approach allowed for isolating *M. sympodialis* and *M. furfur* as separate etiological factors of hyperpigmented and hypopigmented skin lesions, respectively. Interestingly, hypopigmented patches, localized on forearms and in the lower parts of arms (Fig. 1c), showed the yellow to yellow-orange fluorescence in the light of Wood’s lamp. Positive results of examination under Wood’s lamp are present only in about one third of the PV cases [4]. In documented cases of this type [4, 26], *M. furfur* was isolated from such lesions, suggesting this species contributes to the fluorescence detected under the Wood’s lamp illumination. It is well established, based on experimental studies, that only *M. furfur* among all the species of the genus is able to synthesize specific indole compounds, including pityriarubins, malassezin and pityrialactones that, act as fluorochromes [4, 20, 33]. Unlike the macules on forearms and lower parts of the arms, hyperpigmented lesions showed lack of fluorescence under Wood’s lamp and strikingly these regions contained only *M. sympodialis*, while *M. furfur* was not detected.

Although PV never leads to scars and most of currently known etiological factors of this disease are highly susceptible to commonly used antifungal drugs [21], occasionally it takes extended time to restore the skin’s original appearance [17, 36]. Regardless of what antifungal therapy has been applied to treat PV, it is often difficult to assess if the treatment has been effective. This is due to the fact that the dead fungal cells remain on the skin surface for several weeks after the therapy has been completed [18]. In the case study presented here, direct examination of the skin scrapings, which was performed 2 weeks after finished treatment, still revealed the presence of a small number of *Malassezia* cells. On the other hand, attempts to culture collected biological material have confirmed that both etiological factors of PV were killed although pigmentation changes were still present for several weeks, what was consistent with literature [17, 31]. However, these changes gradually subsided over the following weeks. Finally, direct microscopical examination performed 2 months after the therapy has been completed, revealed negative cultures on MLNA medium and confirmed a lack of structures typical for Malassezia. In contrast, control examination of unaffected skin around the neck and face performed after this time, still revealed the presence of *M. restricta* and *M. sympodialis* cells, what was confirmed by positive results of direct examination, as well as it was visible in the mixed culture of both isolates on the MLNA medium. Our case study showed that whereas etiological factors of PV were effectively eradicated from the affected skin locations, in the areas of uninfected skin the normal lipid-dependent mycobiota was still present. Such an outcome was possible thanks to the global approach taking into account not only effective treatment of infection but also protection of microbiota present on healthy skin. With this in mind, the management of infection did not include systemic therapy, but utilized cream and gel, which were applied topically only to affected areas of the skin.

According to recent studies, the most abundant human skin inhabitants are *M. globosa* and *M. restricta* followed by *M. sympodialis* [1, 2, 9, 21]. In our case study, *M. restricta* and *M. sympodialis* were isolated from healthy skin of man and his wife as the most probable components of their normal skin mycobiota. Moreover, *M. sympodialis*, which was isolated as etiological factor of PV from hyperpigmented lesions, was isolated simultaneously from healthy skin around the face and neck of the patient and from the chest and neck of patient’s wife. Nucleotide sequences analysis for *M. sympodialis* isolates obtained both from man and his wife suggested the same strain, at least with respect to nucleotide sequences of the ITS region. During the control examination of women’s skin, *M. furfur* was never isolated. It cannot be excluded that one (or both) of identified etiological factors of PV in the man, typically as single blastoconidia, was (were) earlier normal component(s) of healthy skin mycobiome. Such a possibility is also very likely considering the fact that these species are described elsewhere as components of human skin mycobiome, although not as common as previously mentioned *M. globosa* and *M*. *restricta* [1, 2, 21]. Previously described cases suggest that some endogenous and/or exogenous factors may have occurred, leading to the dysbiosis [6] and development of typical disease symptoms [4, 22]. As it is described elsewhere [4, 5, 17], the disease symptoms usually appear when cell proliferation intensifies and mycelial growth is promoted within the tissue. Our results also point to a blurred borderline between physiological conditions and a pathological state where yeasts overgrow and cause the disease. All the presented herein facts allow to conclude that similar events led to the development of PV in the case studied here. Moreover, improper treatment with fluconazole executed by the patient, before visiting dermatologist, could have contributed to the acquired resistance of *Malassezia* to this drug.

Although, no reference method has been developed for *Malassezia* yeasts susceptibility testing, we decided to follow one of the newest and commonly cited protocols developed by Rojas et al. [37], which is closest to CLSI (Clinical & Laboratory Standards Institute) reference protocol [38]. *Malassezia* lipophilic fungi are generally seen as highly sensitive to commonly used topical and systemic drugs [21, 36]. For this reason, it is generally considered that patients afflicted by PV might be primarily treated with topical agents [19, 36]. However, special care and adequate monitoring should be ensured due to recurrence, which is frequently reported for PV and estimated to occur in *~* 60% and ~ 80% of patients in the first and the second year, respectively [19, 21]. On the other hand, an acquired resistance to fluconazole and other azole drugs [39–41], commonly identified for *Malassezia* spp. in vitro*,* was an important reason to assess the susceptibility profiles of all three *Malassezia* strains. Results of susceptibility testing allowed the dermatologist to choose the best option for therapy. Considering this, the doctor included antifungal drugs representing two different classes i.e. alliloamines (terbinafine) and hydroxypyridones (ciclopirox). These drugs are characterized by entirely different mechanism of action [42, 43]. Moreover, both these drugs have been used successfully against *Malassezia* species and were recommended [44]. While Malassezia yeasts are susceptible to azole-type, hydroxypyridone-type and allylamine-type drugs [21, 36, 45], the last mentioned are not effective given orally [46]. Taking this into consideration and based on the results of susceptibility tests (Table S1, additional file 1), local therapy including ciclopirox and terbinafine was selected. This approach was further justified given our results indicating low susceptibility of *M. furfur* (Mf_MD2 strain to fluconazole (Table S1, additional file 1). To elucidate the most probable mechanism of resistance to fluconazole, additional susceptibility tests were performed (Fig. S1, additional file 1) with rhodamine 6G and daunorubicin (described and discussed in details in,Additional file 1”). While in the case presented here topical treatment was effective and no relapses were observed, cases of severe or recalcitrant PV may still require oral administration of itraconazole or fluconazole (excluding strains resistant to azoles). Problems related to therapy of PV, especially PVa are described elsewhere in more details including various treatment regimens [19].

**Fig. 3:**
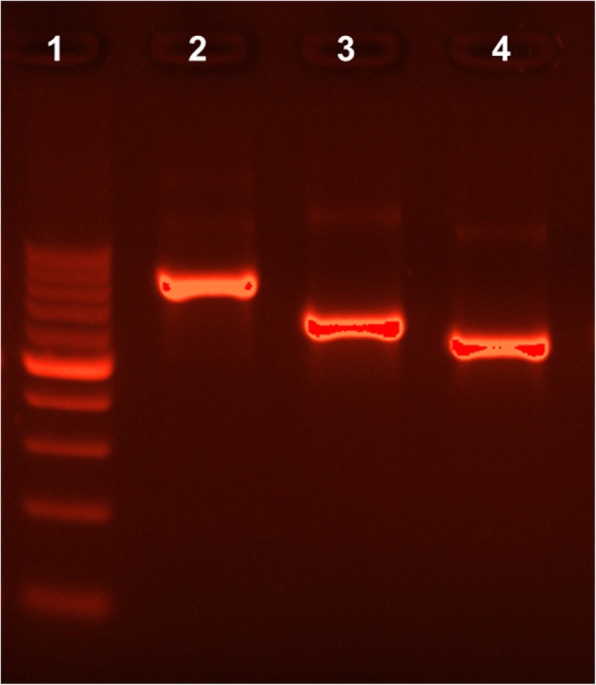
The ITS 1/4 amplification products of *Malassezia* species from pure cultures in 2% agarose gel. Lane 1 - molecular-weight size marker (DNA Marker 1, A&A Biotechnology, range: 100–1000 bp., concentration: 100 ng/μl) Lane 2 - *Malassezia furfur,* Lane 3 - *Malassezia restricta*, Lane 4 - *Malassezia sympodialis*. For all the PCR products (marked as 2–4) 20 ng/μl were loaded per well

Successful therapy in the case presented here was possible thanks to the unambiguous species identification (Table 1, Fig. 3). Herein should be also mentioned that non-culture-based molecular methods, described elsewhere [47, 48] are currently considered the best way to detect and identify Malassezia species from the clinical specimens. However, non-culture-based molecular methods although very effective do not make it possible to state whether fungal specific structures are still viable or already killed by antifungal drug. To overcome this limitation, we decided to perform molecular identification based on pure cultures, which allowed monitoring the progress of antifungal therapy with respect to pathogen viability. For instance, examination performed after the first 2 weeks of therapy revealed that cultures on MLNA medium were negative while Malassezia specific structures were still visible during microscopic direct examination. Furthermore, the unambiguous species identification (Table 1, Fig. 3) allowed us to link the occurrence of hypo- and hyperpigmented skin lesions with separate etiological agents, *M. furfur* and *M. sympodialis*, respectively.

**Table 1:** BLAST analysis of nucleotide sequences of the PCR^a^ products obtained in amplification with ITS1 and ITS4 primers^b^. All E values^c^ were 0.0

Fungal strains isolated			Identity with sequence from GenBank		
Identified species	GenBank Accession No.	Sequence length [bp]^d^	Query Cover,%	Identity,%	Accession
*Malassezia furfur*	MN888953.1	789	100	99.11	KY104128.1
*Malassezia restricta*	MN888954.1	711	82	96.93	AY387143.1
*Malassezia sympodialis*	MN888952.1	621	95	98.82	KY104182.1 and KY104176.1

^a^*PCR* Polymerase chain reaction; ^b^*ITS* Internal transcribed spacer; ^c^E value - BLAST E-value, number of expected hits of similar score that are available just by chance; ^d^*bp* Base pairs

Both species can be a part of healthy skin mycobiome and under specific conditions can simultaneously cause PV [4, 8, 11, 17]. In the context of the infection and its management, *M. furfur* appears to be more problematic than *M. sympodialis*. This is evidenced by serious bloodstream infections so far only described for *M. furfur* [21]. In the context of superficial infections, this predominance may result from better predisposition of *M. furfur* to develop invasive mycelial form, which can penetrate human stratum corneum [5, 12, 15]. Also, unlike *M. sympodialis*, *M. furfur* is naturally able to synthesize specific fluorochromes, including indole derivatives [19, 20] and azelaic acid [32]. Both mentioned compounds have impact on the presentation of PV due to their negative effects on melanocytes and inhibition of the melanogenesis [19, 32–34] that can lead to white discoloration of skin resembling *vitiligo* [19]. Such effects may be the cause of skin discoloration, observed in our patient (Fig. 1c).

**Fig. 4:**
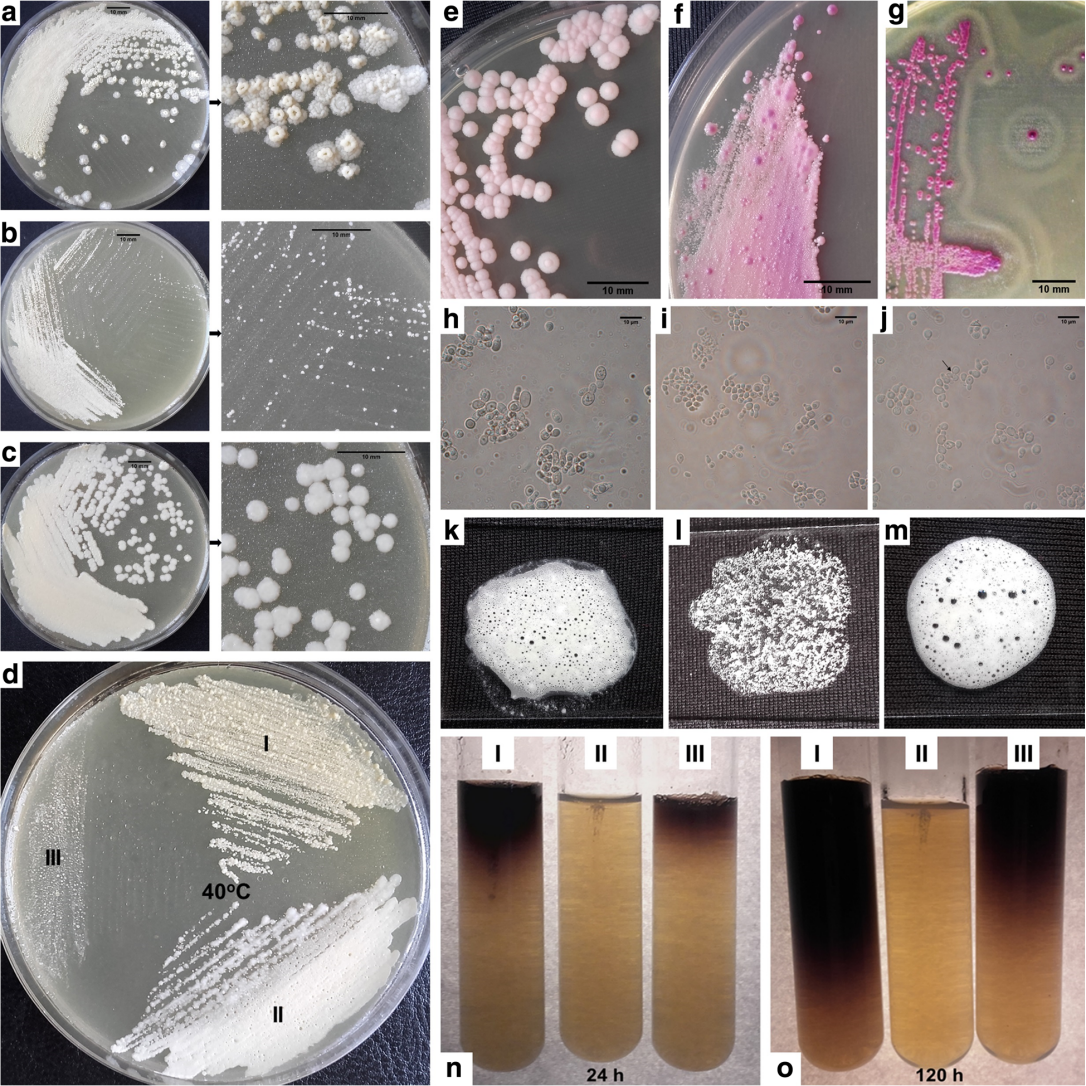
Species identification based on conventional mycological tests. **a**-**c**. Appearance of *Malassezia* species on MLNA medium after five days incubation at 32 °C, **a**. *Malassezia furfur*, **b**. *Malassezia restricta,* and **c**. *Malassezia sympodialis*; (**d**) differences in the ability to grow at 40 °C between isolated strains, I. *Malassezia furfur*, II. *Malassezia sympodialis*, and III. *Malassezia restricta*; (**e**-**g**) appearance of *Malassezia* species on CHROMagar *Malassezia* medium after four days of incubation at 32 °C, **e**. *Malassezia furfur*, **f**. *Malassezia restricta,* and **g**. *Malassezia sympodialis*; (**h**-**j**) micromorphology, typical for each species, observed with DIC microscopy, **h**. *Malassezia furfur*, **i**. *Malassezia restricta,* and **j**. *Malassezia sympodialis,* magnification: (**h**-**j**) 1000x; (**k**-**m**) differences in catalase activity between three isolated *Malassezia* strains. **k**. *M. furfur*, **l**. *M. restricta,* and M. *M. sympodialis*; (**n**, **o**) differences in β-glucosidase activity after 24 h (**n**) and 120 h (**o**) between three isolated *Malassezia* strains, I. *M. sympodialis*, II. *M. restricta,* and III. *M. furfur*. Scale bars correspond to: 10 mm in case of **a**, **b**, **c**, **e**, **f,** **g** and 10 μm in case of **h**, **i**, **j**

The species specific predisposition to synthesize of fluorochromes, that have the ability to absorb light of a certain wavelength and re-emit light at a longer wavelength, is an important element of the differential diagnosis based on the use of Wood’s lamp [4, 20, 26]. Previous studies have indicated that hypopigmented lesions characterized by green-yellowish fluorescence in Wood’s lamp were colonized by *M. furfur*, while hyperpigmented non-fluorescent areas of skin contained other species of the Malassezia genus including *M. sympodialis* [34]. Our study confirmed this relationship with both types of skin areas detected simultaneously in the same patient. *M. sympodialis*, in contrast to *M. furfur*, is characterized by the ability to synthesize melanin [26, 35], which may explain the characteristic appearance of hyperpigmented lesions [26]. In Malassezia diagnostics, the results of conventional tests based on pure cultures are often insufficient for unambiguous species identification. However, tests based on pure cultures presented here (Fig. 4 and,Additional file 1”) provided additional support for molecular techniques and allowed for better understanding of the biology and predisposition for pathogenicity of Malassezia species isolated in this work. We would like to point out that testing utilization of glycine is yet another useful diagnostic method, which while not performed in our studies, may be helpful in future investigations [49].

Our case study has demonstrated that the used topical antifungal drugs, characterized by different mechanisms of action was sufficient to cure PV without any relapses. However, further investigations including large study groups would be desirable. Such additional studies are necessary to shed more light on causative agents of hyperpigmented and hypopigmented cases of PV, especially when the two skin regions are found in the same patient. Moreover, future investigations should further evaluate the proceedings similar to those described here that allowed eradication of the infection while preserving the composition of the microbiome.

## Supplementary information

**Additional file 1 **(DOCX 168 kb) content: METHODS (Direct mycological examination; Culture conditions and conventional mycological tests; Molecular identification; Antifungal susceptibility testing; Microscopy and imaging; Statistical analysis) and RESULTS (Direct examination and observations in the light of Wood’s lamp, Conventional mycological diagnostics, Molecular identification, Susceptibility tests and their interpretation, **Table S1**; **Fig. S1**; Supplementary references).

## Data Availability

All the datasets supporting the conclusions of this article are available and included within the main text of this work and its Additional file 1. Relevant nucleotide sequences and isolated strains are deposited in GenBank under accession numbers: MN888953.1; MN888954.1; MN888952.1 and in the Institute of Genetics and Microbiology, University of Wroclaw, respectively.

## Abbreviations

CLSI: Clinical & laboratory standards institute
MLNA: Modified Leeming-Notman agar
PCR: Polymerase chain reaction
PV: *Pityriasis versicolor*
