# Supplementary material for: A case of anti- pityriasis versicolor therapy that preserves healthy mycobiome
Source: BMC Dermatol. 2020 Sep 29;20:9. doi: 10.1186/s12895-020-00106-x (PMC7526128; doi:10.1186/s12895-020-00106-x)
Supplement: Supplementary file 1 — Additional file 1 (DOCX 168 kb) content: METHODS (Direct mycological examination; Culture conditions and conventional mycological tests; Molecular identification; Antifungal susceptibility testing; Microscopy and imaging; Statistical analysis) and RESULTS (Direct examination and observations in the light of Wood’s lamp, Conventional mycological diagnostics, Molecular identification, Susceptibility tests and their interpretation, Table S1; Fig. S1; Supplementary references). [file 12895_2020_106_MOESM1_ESM.docx]

**Methods**

**Direct mycological examination**

Immediately after dermatological consultation and before the onset of antifungal treatment, direct examination was performed in the local mycological laboratory. Microscopical examinations of skin scrapings were performed in duplicate during two independent visits in laboratory taking place with an interval of one week. Microscopic preparations were made from skin scrapings collected using sterile surgical scalpel and suspended in clearing fluid [1] comprising dimethyl sulphoxide (DMSO) and 10% KOH (Fig. 2A-C, main text). For better visualization skin scrapings were also mounted directly on glass slides according to periodic acid Schiff (PAS) staining technique [1] (Fig. 2D-F, main text). After consultation with dermatologist, it was decided to collect skin scrapings from hyperpigmented and hypopigmented skin lesions separately to sterile Petri dishes (every time using new surgical scalpel). Samples of the scarfskin from healthy skin (without any visible skin lesions) around the neck and face were collected as reference point control. Furthermore, similar control biological material was collected from the chest and neck area of the patient's wife, who never suffered from any dermatological diseases.

**Culture conditions and conventional mycological tests**

*Media for primary isolation and initial differentiation*

Standard mycological procedures and the appropriate culture media were used for primary isolation of fungi from biological material and for the initial differentiation of possible etiological factors of PV. All the samples collected from the skin of patient and his wife were immediately transferred onto Leeming–Notman agar (MLNA) complete medium: 1% peptone, 1% glucose, 0.2% yeast extract, 0.8% ox bile, 1% glycerol, 0.05% glycerol monostearate, 0.5% Tween 60, 2% olive oil, 2% agar (instead 1.5%), pH 6.0, additionally supplemented with 0.025% chloramphenicol and 0.025% ampicillin (our modification) [2]. This medium was used for the primary isolation. The MLNA plates were warmed to room temperature prior to inoculation to avoid thermal shock of cells. Plates, covered by parafilm to avoid medium drying after inoculation of skin scrapings, were incubated up to 10 days at 32^o^C. This medium was also used to obtain pure cultures of each strain, based on the streak-plate technique [3], and to evaluate colony morphology (Fig. 3A-C, main text). The first colonies appeared on complete MLNA medium just after a few days and were systematically isolated on the new plate. Pure cultures were used to perform all further experiments and to assess the macro- and micromorphology of each obtained isolate. In the case of skin scrapings collected from hypopigmented and hyperpigmented skin lesions, colonies were observed after four to five days of incubation. Finally, for the material collected from healthy skin, colonies were visible up to two days later. Differentiation of isolates from other lipid independent fungi [4] was performed based on standard SDA medium, pH 5.6 (Sabouraud 4% dextrose agar, Merck KGaA, Germany) supplemented with 0.005% chloramphenicol and 0.01% gentamicin and synthetic YNBA medium without lipids, as previously described [4]. Additionally, CHROMagar *Malassezia* plates (CHROMagar, Paris, France; 1.5% agar, 3.8% peptones and extracts, 0.05% chloramphenicol, 0.28% chromogenic mix, 0.2% glycerol and 1% Tween 40, pH 6.3 ± 0.3) [5] were used to determine the ability to precipitate in the presence of Tween 40 and to assess species dependent character of growth [6]. CHROMagar *Malassezia* plates allow differentiation between *M. sympodialis*-related species and species outside this group [7]. All plates were incubated aerobically at 32^o^C for 4 or 5 days (Fig. 3A-C and E-G, main text).

*Assessment of growth at 40^o^C*

All isolated *Malassezia* strains were assessed for the ability to grow at 40^o^C [8]. MLNA plates were inoculated and incubated up to 5 days. Growth, confluent or as single colonies, was evaluated visually and photographs were taken after total incubation period (Fig. 3I, main text). The second plate was incubated at 32^o^C as control (Fig. 3III, main text).

*Determination of the catalase activity*

Assessment of catalase activity [2] was determined by adding a drop of 20% hydrogen peroxide onto a smear of fresh culture spread on a microscopic glass. Results of tests were read visually. Photographs were taken immediately after spotting the reagent onto the culture smear (Fig. 3K-M, main text).

*Evaluation of growth on esculin medium*

The β-glucosidase activity was assessed for cultures set up on esculin agar (EA) comprising: 1% bacteriological peptone, 0.1% ferric ammonium citrate, 0.15% esculin and 2% agar (instead 1,5%), pH 7.4. EA medium was prepared as described[2] and distributed in glass tubes. Medium in tube was inoculated by stabbing the pure *Malassezia* culture into the agar using an inoculation needle. Inoculated media were incubated at 37^o^C and examined daily for five days [2]. Photographs were taken after the first and fifth day of incubation (Fig. 3N, O, main text).

**Molecular identification**

*Genomic DNA extraction*

DNA was extracted from pure cultures previously obtained from a single colony. For genomic DNA isolation, *Malassezia* spp. strains were cultivated on MLNA medium [2] under aerobic conditions at 32^o^C for 96 hours. Cellular DNA was extracted based on Rose et al. protocol [9] adopted with some modifications (Dyląg M., described herein) to improve efficiency of nucleic acids isolation from lipid-rich thick cell wall [10] of *Malassezia* spp. cells. Briefly, cells were harvested from agar surface using sterile cotton swabs (Equimed) and resuspended in sterile nanopure water followed by centrifugation (1 min, 14,000 rpm, room temperature). Next, cell pellets were resuspended in 200 µl of cell wall digestion buffer (freshly prepared) containing 0.5% zymolyase (20,000 U/ml) in 50 mM Tris-HCl buffer (pH 7.5), 25 mM EDTA and 1% β-mercaptoethanol. Samples were incubated for 30 min at 37^o^C with horizontal shaking at 1400 rpm (Eppendorf ThermoMixer® C). During this time samples were mixed few times by inverting the Eppendorf tubes. Next, 200 µl of fresh lysis solution, obtained by mixing equal volumes of 0.4 M NaOH and 2% SDS, was added followed by 20 µl of Proteinase K (20 mg/ml). Samples were incubated for 10 min at 60^o^C with horizontal shaking (as previously). After incubation, 200 µl of 3 M sodium acetate (pH 5.2) was added and mixed vigorously, followed by 20 min incubation at -20^o^C and then centrifugation (14,000rpm, 4^o^C, 15 min). The supernatant was carefully transferred to a new microcentrifuge tube and mixed by inverting with equal volume of cold (-20^o^C) isopropanol. The precipitated DNA was collected by centrifugation (14,000 rpm, 4^o^C, 15 min) and washed two times with 70% ethanol (0.5 ml, -20°C), dried on a vacuum dryer (speedvac) and resuspended in 20-50 µl of sterile nanopure water (DNAse free) with RNAse (300 µg/ml). Obtained genomic DNA was purified using Clean-Up kit (A&A Biotechnology), as per the manufacturer’s instructions and used as template for PCR reaction. Purity and concentration was checked spectrophotometrically (NanoPhotometer^®^ NP80, Implen GmbH).

*Identification of Malassezia spp. strains using molecular tools*

Molecular speciation was based on sequencing of the PCR product obtained with ITS1/4 pair of primers complementary to the rDNA gene cluster, as defined by White et al. [11]. DNA amplification was performed in a 50 μl volume using the two-fold concentrated PCR mixture containing a Taq polymerase (0.1 U/ml), dNTP mix (0.5 mM of each), MgCl_2_ (4 mM) (A&A Biotechnology, Poland), 0.25 μM of ITS1/4 primers (Genomed, Poland). The oligonucleotide sequences, described elsewhere [11], were as follows: 5`-TCCGTAGGTGAACCTGCGG-3` (ITS1) which hybridizes at the end of 18S rDNA and 5`-TCCTCCGCTTATTGATATGC-3` (ITS4) which hybridizes at the beginning of 28S rDNA [11]. Genomic DNA, at final concentration 100 ng per 50 μl, served as template. PCR reaction was performed in the BIO-RAD T100™ thermal cycler. Initial denaturation for 5 min at 94°C was followed by a single cycle comprised of 30 s denaturation at 94°C, 30 s annealing at 55°C, 45 s elongation at 72°C, and finally followed by elongation for 7 min at 72°C at the end of 35 cycles. All the PCR products were separated on agarose gel (2%) and visualized using the Gel Doc EZ System (BIO-RAD). PCR products (Fig. 4, main text) were purified from the agarose gel using Clean-Up kit (A&A Biotechnology, Poland), diluted to concentration equal to 50 ng/µl and sequenced in two directions with the same primers as used in PCR reaction by the Sequencing Service at Macrogen (The Netherlands, http://dna.macrogen.com/eng/, RRID:SCR:014454).

The sequences of PCR products were analyzed using BioEdit Sequence Alignment Editor (http://www.mbio.ncsu.edu/bioedit/bioedit.html, RRID:SCR:007361) and compared (Table 1, main text) with those deposited in the GenBank of the National Center for Biotechnology Information (NCBI, Bethesda, MD, USA, RRID:SCR:002760) using the BLAST algorithm (RRID:NCBIGene:12506). For additional confirmation of species affiliation of three *Malassezia* spp. strains homology of nucleotide sequences was analyzed based on CLUSTALW program (https://www.genome.jp/tools-bin/clustalw, RRID:SCR:002909). CLUSTALW allows for the analysis of the genetic similarity between studied *Malassezia* strains based on ITS sequences of other fungi deposited in the NCBI database. Homology to reference strains with nucleotide sequences deposited in GenBank was also assessed based on constructed phylogenetic trees (data not shown). The genetic distances between analyzed sequences were assessed using the likelihood method [12]. Phylogenetic trees were constructed using a neighbor-joining (NJ) technique and confirmed by bootstrap method with 1000 repetitions [13].

**Antifungal susceptibility testing**

*In vitro* antifungal susceptibility of isolated *Malassezia* strains was determined (Table S1) by the reference Clinical & Laboratory Standards Institute (CLSI) broth microdilution procedure [14] according to protocol M27-A3, which is a version of the method utilized for yeast-like fungi that has been modified for working with lipid-dependent opportunistic pathogens [15]. All chemicals utilized in this method were, if not stated otherwise, purchased from Sigma-Aldrich (Poland) and were of analytical grade. The antifungal agents and concentrations were as follows: 0.002 - 16 µg/ml for ciclopirox (CIC), terbinafine (TER), itraconazole (ITZ), miconazole (MCZ) and ketoconazole (KTZ), and 0.25 - 256 µg/ml for fluconazole (FCZ). Antifungal stock solutions were prepared in DMSO, except for CIC and TRB, which were dissolved in ethanol (POCH, Poland). Additionally, rhodamine 6G (R6G), daunorubicin, and tacrolimus (FK506), all purchased from Sigma-Aldrich (Poland), were used for some susceptibility tests as a control supporting the resistance phenotype detection [16-20]. Stock solutions for R6G and daunorubicin were prepared in methanol at a final concentration equal to 40 mg/ml (83.5 mM) and 30 mg/ml (56.9 mM), respectively. Stock solution for FK506 was prepared in ethanol at 25 mg/ml (31 mM). Before the susceptibility testing, each isolate was incubated for five days at 32°C on MLNA medium to ensure its viability in culture. The size of inoculum suspensions was verified by viable colony counts on MLNA complete medium. *Candida parapsilosis* ATCC 22019 and *Candida krusei* ATCC 6258, obtained from American Type Culture Collection, were used as quality control strains to check the reproducibility of the results. All isolates were tested in triplicate on different days. Growth and sterility control wells were also included. Inoculated microplates were incubated in humidity chambers to avoid desiccation. Assay microtiter plates with 96 U wells were incubated for four days at 32°C and then read spectrophotometrically using Varioskan LUX multimode microplate reader (ThermoFisher) at 530 nm wavelength (λ). Only if two replicates displayed identical results was the fungal strain given the final MIC value of the antifungal preparation tested. The endpoint for minimal inhibitory concentration (MIC_90_) was the antifungal concentration at which prominent inhibition of growth, i.e. ≤ 90% as compared to that of the control was observed [21].

**Microscopy and imaging**

Microscopic observations (Fig. 3H-J, main text) were performed using differential interference contrast (DIC) microscopy (Zeiss microscope, Axiovert 200M, Zeiss ID#M 202086, Carl Zeiss MicroImaging, Inc., Thornwood, NY, USA) with built-in camera for photographic documentation (AxioCam HRm) and interfaced with AxioVision Rel 4.8 software (Carl Zeiss, Thornwood, NY, RRID:SCR:002677). Microscopic preparations in case of direct examination of skin scrapings (Fig. 2A-C and 3A-C, main text) were examined under light microscopy (OPTA-TECH, model: MB200) with built-in camera for photographic documentation (OPTA-TECH, 3MP) and interfaced with OptaView-IS software (OptaView-IS, version 3.6.6). For the evaluation of the cell body diameter, cells were measured based on the images using ImageJ (https://imagej.nih.gov/ij/, RRID:SCR:003070).

**Statistical analysis**

The susceptibility test data were subjected to statistical analysis using the Statistica 13.3 package (StatSoft Polska Sp. z o.o., Poland, RRID:SCR:014213). For this purpose, one-way analysis of variance (ANOVA) and the Tukey HSD (honest significant difference) test were utilized. The MIC values, were not statistically different at P-value < 0.05 according to Tukey’s HSD test. Prior to ANOVA, the data were transformed to Bliss [22] angular degrees by applying the formula y = arcsin (value)−0.5. After transformation, the variance was approximately constant, allowing ANOVA to compare particular components [22] Additionally, the results of susceptibility tests were presented as the mean ± SD from of at least three independent experiments (three replicates).

**Results**

**Direct examination and observations in the light of Wood’s lamp**

Preparations made from skin scrapings collected both from hyper- as well as from hypopigmented skin lesions showed the abundance of unipolarly budding blastoconidia and pseudohyphae in the characteristic “spaghetti and meatballs” pattern (Fig. 2A, C and Fig. 2D, F, main text). It should be also noted that in skin scrapings collected from hypopigmented skin lesions pseudohyphae dominated over budding cells (Fig. 2D, main text). In contrast, direct examination of fragments of the epidermis taken from healthy skin areas of the neck and face revealed only the presence of single and dispersed monopolarly budding cells with thick cell wall. Those cells were clearly visible against the background of keratinocytes (Fig. 2B and Fig. 2E, main text). Additionally, in the light of Wood’s lamp, fluorescence characteristic for PV was observed. A dermatoscope was also used to provide light, magnification and illuminate morphology of skin lesions. It should be emphasized that only skin lesions localized on forearms and at the bottom of the arms gave strong yellow, yellow-green to yellow-orange fluorescence, while for hyperpigmented macules located in other areas of the body no fluorescence was observed. A similar pattern was found in case of patient`s wife, where direct examination (performed for control purposes) of healthy skin of the neck and face revealed only single yeast cells and no fluorescence in light of Wood’s lamp was detected.

**Conventional mycological diagnostics**

Yeast isolates from three different body sites presented generally three different macromorphological (Fig. 3A-C, main text) and micromorphological (Fig. 3H-J, main text) types. The isolate (named as ,,isolate 1”, finally identified as *M. furfur*) cultured from skin scrapings of hypopigmented lesions after five days on MLNA medium formed large opaque to white colonies, 3-6 mm in diameter. All the colonies were irregular with lobate margin and in the context of surface texture smooth with clear convex (Fig. 3A, main text). Micromorphology was characterized by cylindrical to ellipsoidal cells varied in size with typically one broad base bud. It should be mentioned that this strain presented the largest cells, up to 6-8 µm (Fig. 3H, main text). In contrast, colonies of isolate (named as ,,isolate 2”, finally identified as *M. sympodialis*) cultured from hyperpigmented lesions although equally large (4-7 mm in diameter) were typically smooth, flat, white to pale cream, and clearly butyrous. The margin of colonies was only slightly lobate (Fig. 3C, main text). Colonies of this isolate were easily resuspended in water or liquid media. In contrast to isolate 1, isolate 2 was characterized by smaller cells up to 3.5-4 µm, globose to ovoid, usually with one monopolar or sometimes with buds emerging sympodially (Fig. 3J, black arrow, main text). The third isolate (named as ,,isolate 3”, finally identified as *M. restricta*), cultured from diagnostic material collected from healthy skin areas, was characterized by a growth rate much slower than the other two and formed the smallest colonies, 1-2 mm in diameter. The colonies of isolate 3 were punctiform, slightly rough, hard and brittle with irregular margin (Fig. 3B, main text). In the context of micromorphology, the cells of isolate 3 were the smallest among all three isolates, with the dimensions up to 2.5-3 µm. Isolate 3 cells were globose to ovoid with only one small bud on one of the poles of the cell (Fig. 3I, main text).

None of the three isolates grew on standard Sabouraud dextrose agar (SDA) medium, or synthetic yeast nitrogen base agar (YNBA) medium at 32^o^C, consistent with lipid-dependent growth requirement. Initial differentiation of the strains was possible based on CHROMagar *Malassezia* (Fig. 3E-G, main text). On this medium, only the culture of the isolate 2, obtained from hyperpigmented skin lesions, developed crystal precipitates diffusing into the agar in direct contact with the colonies (Fig. 3G, main text). This phenomenon was newer observed in case of two other isolates (Fig. 3E, F, main text). The results of this simple experiment allowed us to conclude that isolate 2 represented *M. sympodialis,* which was able to precipitate Tween 40, while the other two isolates did not belong to this group. Moreover, the macromorphology of colonies formed on this medium by isolate 1, pale pink, large and wrinkled, suggested *M. furfur* (Fig. 3E, main text). Results of the other tests, as part of conventional mycological diagnostics, shed more light on identification of isolates up to species level. For these purposes, evaluation of β-glucosidase activity, an assessment of growth at 40^o^C, and the test for catalase activity were carried out. Results of all three experiments were persuasive and especially in case of isolate 3 clearly indicated *M. restricta*. This isolate was not able to grow at 40^o^C (Fig. 3III, main text) and showed lack of catalase (Fig. 3L, main text) and β-glucosidase activity (Fig. 3NII and 3OII, main text). Unlike isolate 3, isolates 1 and 2 grew very well at 40^o^C (Fig. 3I, II, main text). Both isolate 1 and 2 showed strong catalase reaction (Fig. 3K, M, main text) and were able to break down β-glycosidic bonds in the esculin molecule (Fig. 3NI, III and 3OI, III, main text) although this activity was stronger in case of isolate 2 (clearly visible after 24h, Fig. 3NI, main text).

**Molecular identification**

Although the results of conventional mycological diagnostics were very useful and self-explanatory they did not allow for unequivocal species identification. Therefore, it was necessary to include molecular diagnostics techniques. Genomic DNA, isolated from each of three isolates, was used as template in amplification reaction with ITS1/ITS4 primers. For each isolate only one single PCR (polymerase chain reaction) product was obtained (Fig. 4, main text). The molecular sizes of obtained amplicons for isolate 1, 2 and 3 were 789, 621, and 711 bp, respectively. PCR products were sequenced and compared against the GenBank database using the Basic Local Alignment Search Tool (BLAST). Nucleotide sequences of isolate 1, 2, and 3 shared 96.93-99.11% identity and the closest match was found with the corresponding sequences of *M. furfur* CBS 7043, *M. sympodialis* 152.1 and *M. restricta* CBS 7877, respectively (Table 1, main text). In all of the BLAST analyses the results were highly significant (E values 0.0). Importantly, the results of molecular identifications were consistent with identifications derived from conventional mycological methods.

The nucleotide sequences of isolate 1, 2 and 3, named as *M. furfur* (Mf_MD2), *M. sympodialis* (Msy_MD10) and *M. restricta* (Mr_MD2), were deposited in the GenBank database under the accession numbers MN888953.1, MN888952.1, and MN888954.1, respectively. All the isolates are stored at local institutional culture collection at the Institute of Genetics and Microbiology, University of Wroclaw, at -80^o^C as cell suspensions in modified Leeming-Notman broth (MLNB) supplemented with sterile 25% glycerol (Chempur, Poland).

**Susceptibility tests and their interpretation**

Among the three isolates, *M. furfur* (Mf_MD2 strain) exhibited the lowest susceptibility toward tested drugs (Table S1). This *M. furfur* strain exhibited particularly low sensitivity towards fluconazole with MIC value equal to 95 µg/ml. Susceptibility of this strain to other azole drugs i.e. itraconazole, ketoconazole and miconazole also appeared to be clearly lower, as compared to other two isolates, with MIC values of 0.125 µg/ml for the first two mentioned and 8 µg/ml for the last one. In contrast to this strain, *M. sympodialis* (Msy_MD10 strain) and *M. restricta* (Mr_MD2 strain) showed relatively higher susceptibilities toward all the tested azole drugs with MIC values 2 µg/ml for fluconazole and miconazole, and 0.03 µg/ml in case of itraconazole and ketoconazole. Apart from azole drugs all the isolates were highly susceptible to terbinafine and ciclopirox. The MIC values of ciclopirox were equal to 0.06 µg/ml in case of *M. furfur* (Mf_MD2 strain) and *M. sympodialis* (Msy_MD10 strain) and 0.08 µg/ml in case of *M. restricta* (Mr_MD2 strain). For terbinafine, MIC values (Table S1) were equal to 1.0 µg/ml in case of *M. restricta* (Mr_MD2 strain) and *M. sympodialis* (Msy_MD10 strain), and 2.0 µg/ml in case of *M. furfur* (Mf_MD2 strain).

Additional susceptibility tests with rhodamine 6G, daunorubicin, and tacrolimus (Fig. S1) revealed that *M. furfur* (Mf_MD2 strain) exhibited clearly lower susceptibility to these compounds, as compared to other two isolates. MIC values for rhodamine 6G and daunorubicin were equal to 40 ± 2.0 µg/ml (83.5 µM) and 30 ± 2.0 µg/ml (56.9 µM), respectively. In contrast, MIC values for R6G and daunorubicin for *M. furfur* CBS7019 reference strain were equal to 2 ± 0.2 µg/ml (4.18 ± 0.42 µM) and 3.0 ± 0.5 µg/ml (5.69 ± 0.95 µM), respectively. The other two *Malassezia* isolates showed equally high susceptibility to R6G and daunorubicin as the reference strain. For rhodamine 6G the MIC values for *M. sympodialis* and *M. restricta* were equal to 1 ± 0.1 µg/ml (2.09 ± 0.21 µM) and 2 ± 0.1 µg/ml (4.18 ± 0.21 µM), respectively. With respect to daunorubicin the MIC values for these strains were equal to 3.5 ± 0.1 µg/ml (6.64 ± 0.19 µM) for both strains. Interestingly, addition of tacrolimus (FK506), which is a well-known calcineurin inhibitor [20] at a final concentration of 5 µmol (4.02 µg/ml) in combination with R6G, daunorubicin, or fluconazole, led to an overall decrease of the respective MIC values (Fig. S1). Specifically, in the presence of FK506 the MIC values for R6G, daunorubicin and fluconazole were decreased 20-, 12-, and 14.6-fold, respectively. It should be mentioned herein, that in control tests FK506, when used alone, showed no inhibitory activity toward any *Malassezia* tested strains in concentrations up to 25 µmol (20.1 µg/ml).

R6G and daunorubicin are well known substrates for ABC proteins, including Pdr5p and Cdr1p present in *Saccharomyces cerevisiae* and *Candida albicans*, respectively, which are responsible for active efflux of toxic substances from the cell [16-19]. Among three *Malassezia* isolates only *M. furfur* showed clearly lowered susceptibility to 6G and daunorubicin. FK506 has been utilized as chemosensitizer to overcome resistance generated by Pdr5p and its homologs, as described elsewhere [18, 19]. As FK506 allowed to significantly restore the susceptibility to R6G, daunorubicin and fluconazole (Fig. S1), these results indirectly suggest that observed resistance phenotype is resulting from overexpression of membrane transporter proteins. This hypothesis will be the subject of subsequent studies.

**Table S1.** Antifungal drug susceptibility profiles of three *Malassezia* strains isolated from skin of man.

| **Species** | **MIC^†^ values** in µg/ml ± SD^§^ values (µM) | | | | | |
| --- | --- | --- | --- | --- | --- | --- |
|  | **CIC^‡^** | **FCZ** | **ITZ** | **KTZ** | **MCZ** | **TER** |
| *Malassezia furfur*  (Mf_MD2) | 0.06 ± 0.002^§^  a^¶^  (0.29 ± 0.01) | 95.0 ± 5.0  a  (310.17 ± 16.33) | 0.125 ± 0.005  a  (0.177 ± 0.007) | 0.125 ± 0.005  a  (0.235 ± 0.009) | 8.0 ± 0.5  a  (19.22 ± 1.2) | 2.0 ± 0.1  a  (6.86 ± 0.343) |
| *Malassezia restricta*  (Mr_MD2) | 0.08 ± 0.004  b^¶^  (0.39 ± 0.02) | 2.0 ± 0.1  b  (6.53 ± 0.33) | 0.03 ± 0.002  b  (0.04 ± 0.003) | 0.03 ± 0.004  b  (0.056 ± 0.007) | 2.0 ± 0.4  b  (4.81 ± 0.96) | 1.0 ± 0.05  b  (3.43 ± 0.07) |
| *Malassezia sympodialis*  (Msy_MD10) | 0.06 ± 0.002  a  (0.29 ± 0.01) | 2.0 ± 0.1  b  (6.53 ± 0.33) | 0.03 ± 0.004  b  (0.04 ± 0.006) | 0.03 ± 0.006  b  (0.056 ± 0.011) | 2.0 ± 0.2  c  (4.81 ± 0.48) | 1.0 ± 0.1  c  (3.43 ± 0.343) |

**^†^**MIC_90%_ – Minimal Inhibitory Concentration of drug for which prominent inhibition of growth and cellular proliferation, i.e. ≥ 90% inhibition was observed, compared to that of the growth control, mean values were calculated based on three independent repetitions ± standard deviation; **^‡^**abbreviations of generic names of antifungals: CIC, ciclopirox; FCZ, fluconazole; ITZ, itraconazole; KTZ, ketoconazole; MCZ, miconazole; TER, terbinafine; ^§^SD – standard deviation values; ^¶^For each MIC values (given in the same column), means followed by the same letter are not statistically different at P-value < 0.05 according to Tukey’s HSD test. Lowercase letters indicate the similarities and differences between three of *Malassezia* strains in terms of susceptibility to given antifungal drug, they refer to column means.

**Fig. S1** Susceptibility of *Malassezia furfur** to fluconazole^†^, rhodamine^§^ and daunorubicin used solely or in combination^‡^


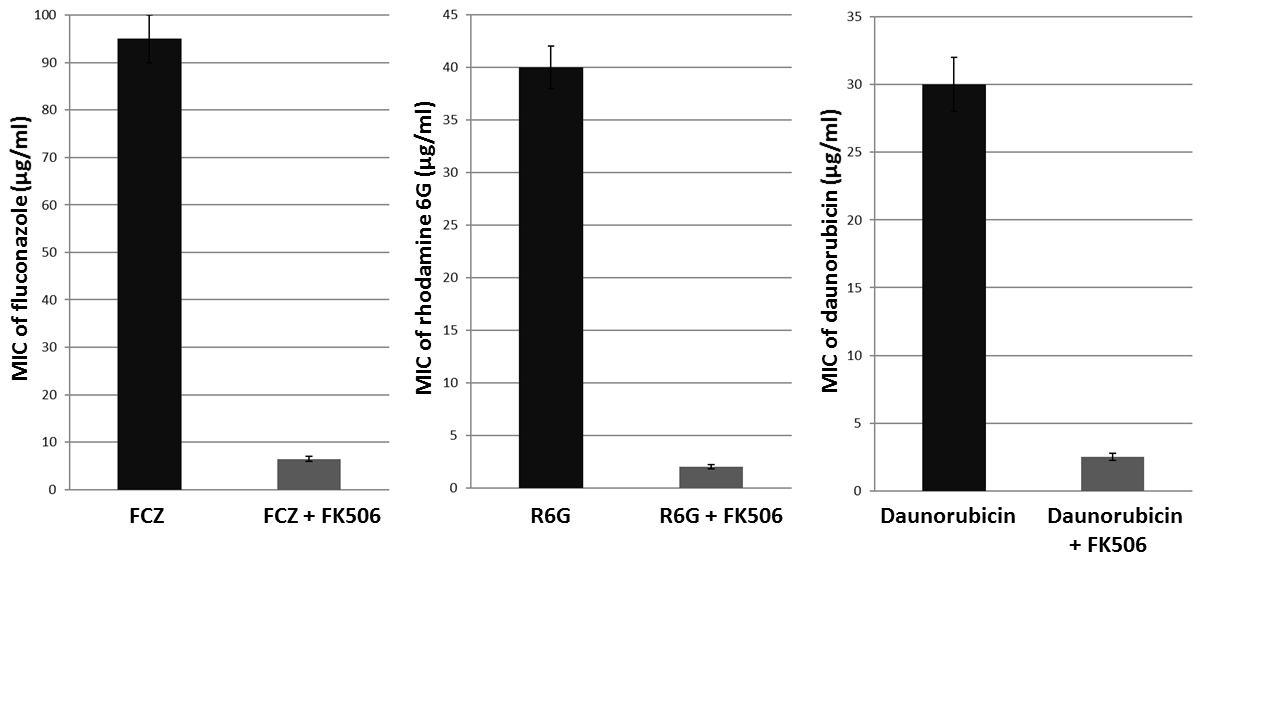


(Legend): **Malassezia furfur* (Mf_MD2 strain), †Fluconazole (FCZ), **^§^**Rhodamine 6G (R6G), ^‡^with tacrolimus (FK506, 5µM).

**SUPPLEMENTARY REFERENCES**

1. McGinnis MR. Laboratory Handbook of Medical Mycology. New York: Academic Press Inc.; 1980. 132p.
2. E. Guého-Kellermann, T. Boekhout, D. Begerow, Biodiversity, Phylogeny and Ultrastructure, In: *Malassezia* and The Skin: Science and Clinical Practice. Springer-Verlag, Berlin Heidelberg, 2010.
3. Sanders ER. Aseptic laboratory techniques: plating methods. J Vis Exp. 2012;(63):e3064.
4. Puig L, Bragulat MR, Castellá G, Cabañes FJ. Characterization of the species *Malassezia pachydermatis* and re-evaluation of its lipid dependence using a synthetic agar medium. PLoS One. 2017;12:e0179148.
5. CHROMagarTM Malassezia, Instructions for use NT-EXT-029 version 6.0. http://www.chromagar.com/fichiers/1574083673NT_EXT_029_V.6.0.pdf Accessed February 8, 2020
6. Kaneko T, Makimura K, Sugita T, Yamaguchi H. Tween 40-based precipitate production observed on modified chromogenic agar and development of biological identification kit for *Malassezia* species. Med Mycol. 2006;44:227-231.
7. Cabañes FJ, Hernández JJ, Castellá G. Molecular analysis of *Malassezia sympodialis*-related strains from domestic animals. J Clin Microbiol. 2005;43:277-283.
8. Aspiroz C, Ara M, Varea M, Rezusta A, Rubio C. Isolation of *Malassezia globosa* and *M. sympodialis* from patients with pityriasis versicolor in Spain. Mycopathologia. 2002;154:111-117.
9. Rose MD, Winston F, Hietr P. Methods in Yeast Genetics. A Laboratory Course Manual. New York: Cold Spring Harbor Laboratory Press; 1990. 198 pp.
10. Mayser P, Gaitanis G. Physiology and Biochemistry, In *Malassezia* and The Skin: Science and Clinical Practice. Berlin Heidelberg: Springer-Verlag; 2010:121-137.
11. White TJ, Burns T, Lee S, Taylor JW. Amplification and direct sequencing of fungal ribosomal RNA genes for phylogenetics, In PCR Protocols: a Guide to Methods and Applications. San Diego: Academic Press; 1990. 315p.
12. Tamura K, Nei M, Kumar S. Prospects for inferring very large phylogenies by using the neighbor-joining method. Proc Natl Acad Sci U S A. 2004;101:11030-11035.
13. Saitou N, Nei M. The neighbor-joining method: A new method for reconstructing phylogenetic trees. Mol Biol Evol. 1987;4:406-425.
14. CLSI. Reference Method for Broth Dilution Antifungal Susceptibility Testing of Yeast; Approved Standard, 3rd Edn. CLSI document M27-A3. Wayne, PA: Clinical and Laboratory Standards Institute, 2008.
15. Rojas FD, Sosa Mde L, Fernández MS, Cattana ME, Córdoba SB, Giusiano GE. Antifungal susceptibility of *Malassezia furfur*, *Malassezia sympodialis*, and *Malassezia globosa* to azole drugs and amphotericin B evaluated using a broth microdilution method. Med Mycol. 2014;52:641-646.
16. Rogers B, Decottignies A, Kolaczkowski M, Carvajal E, Balzi E, Goffeau A. The pleitropic drug ABC transporters from *Saccharomyces cerevisiae*. J Mol Microb Biotech. 2001;3:207-214.
17. Lamping E, Monk BC, Niimi K, et al. Characterization of three classes of membrane proteins involved in fungal azole resistance by functional hyperexpression in *Saccharomyces cerevisiae*. Eukaryot Cell. 2007;6:1150-1165.
18. Guo X, Li J, Wang T, et al. A mutation in intracellular loop 4 affects the drug-efflux activity of the yeast multidrug resistance ABC transporter Pdr5p. PLoS One. 2012;7:e29520.
19. Tanabe K, Bonus M, Tomiyama S, et al. FK506 Resistance of *Saccharomyces cerevisiae* Pdr5 and *Candida albicans* Cdr1 Involves Mutations in the Transmembrane Domains and Extracellular Loops. Antimicrob Agents Ch. 2018;63:e01146-18.
20. Shirazi F, Kontoyiannis DP. The calcineurin pathway inhibitor tacrolimus enhances the in vitro activity of azoles against *Mucorales* via apoptosis. Eukaryot Cell. 2013;12:1225-34.
21. Velegraki A, Alexopoulos EC, Kritikou S, Gaitanis G. Use of fatty acid RPMI 1640 media for testing susceptibilities of eight *Malassezia* species to the new triazole posaconazole and to six established antifungal agents by a modified NCCLS M27-A2 microdilution method and Etest. J Clin Microbiol. 2004;42:3589-3593.
22. Bliss CT. The method of probits. Science. 1934;79:38-39.
